# piRNA-Guided Transposon Silencing and Response to Stress in *Drosophila* Germline

**DOI:** 10.3390/v16050714

**Published:** 2024-04-30

**Authors:** Samantha Ho, William Theurkauf, Nicholas Rice

**Affiliations:** Program in Molecular Medicine, University Campus, University of Massachusetts Chan Medical School, Worcester, MA 01655, USA; william.theurkauf@umassmed.edu

**Keywords:** piRNA, transposon, P-element, transcriptional silencing, DNA damage, heat stress

## Abstract

Transposons are integral genome constituents that can be domesticated for host functions, but they also represent a significant threat to genome stability. Transposon silencing is especially critical in the germline, which is dedicated to transmitting inherited genetic material. The small Piwi-interacting RNAs (piRNAs) have a deeply conserved function in transposon silencing in the germline. piRNA biogenesis and function are particularly well understood in *Drosophila melanogaster*, but some fundamental mechanisms remain elusive and there is growing evidence that the pathway is regulated in response to genotoxic and environmental stress. Here, we review transposon regulation by piRNAs and the piRNA pathway regulation in response to stress, focusing on the *Drosophila* female germline.

## 1. Transposons

In 1950, Barbara McClintock described “mutable loci”, which gave rise to high rates of observable phenotypic variation resulting from the chromosome breakage–fusion–bridge cycle and inferred that this variation was the result of the insertion of genetic elements near the affected loci [1]. These genetic elements are now known as transposons. Transposons were initially considered “junk” DNA or strictly selfish parasitic elements, which utilize the host machinery to replicate and “jump” into new genomic locations, causing potentially deleterious mutations. However, it is now clear that transposons can drive evolution by providing critical regulatory elements and producing beneficial mutations [2,3]. 

Since McClintock’s initial observations, transposons have been discovered in almost every organism across all kingdoms [4,5]. These mobile elements also show a remarkable diversity within the genomes of individual organisms. For example, *Drosophila melanogaster* (*D. melanogaster*) contains over 100 transposon families that make up between 5 and 15 percent of the genome of different strains [6,7]. Additionally, the transposon landscape within a species can be dynamic, as new elements can invade the germline. For example, P-elements invaded *D. melanogaster* within the past 100 years, likely through a jump from *Drosophila willistoni* [8]. Supporting this hypothesis, P-element sequences in these highly divergent species differ by only one nucleotide. Therefore, transposons represent dynamic genome constituents with the potential to induce deleterious mutations or beneficial genetic diversity.

### 1.1. Transposon Classification

**Figure 1:**
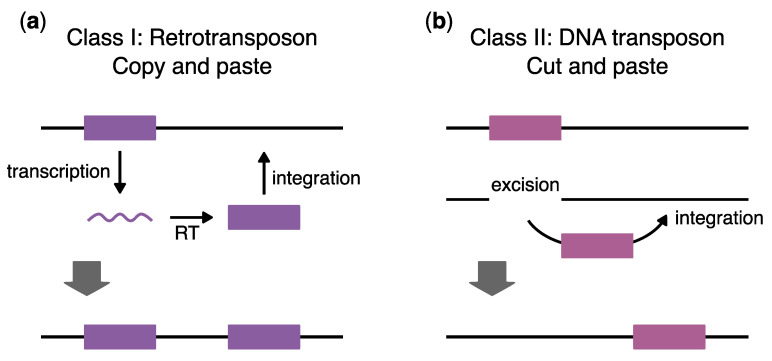
The two classes of transposons: (**a**) Class I retrotransposons use the “copy and paste” mechanism of transposition. These transposons are first transcribed into RNA, reverse transcribed (RT) into DNA, and then integrated into a new location. This results in an increase in transposon copy number. (**b**) Class II DNA transposons use the “cut and paste” mechanism of transposition. These transposons are excised out of their original location and can then be inserted into a new location. This results in double-stranded DNA breaks and does not increase copy number for that transposon.

There are two major classes of transposons, Class I Retrotransposon and Class II DNA transposons, which differ in their mechanism of movement (Figure 1). Class I Retrotransposons move using a “copy and paste” mechanism [4,9] (Figure 1a). In this process, the host machinery transcribes the transposon insertions to produce an RNA intermediate. In autonomous elements, the resulting transcripts encode a reverse transcriptase, which converts the RNA transcript into a DNA copy, and an integrase, which inserts the DNA copy into a new genomic location. This “copy and paste” mechanism results in the duplication of the original transposon at a novel site [9]. 

The LTR subclass of retrotransposons is closely related to exogenous retroviruses. These transposons contain three open reading frames (ORFs) coding for a capsid protein (gag), a polymerase/integrase (pol), and a virus-like envelope (env) [4]. Retrotransposons that contain all three open reading frames can therefore form viral particles and move horizontally, and they are termed endogenous retroviruses [10,11]. For example, the *Gypsy* retrotransposon makes viral particles in the somatic follicle cells that infect neighboring germ cells [12,13]. A second retrotransposon subclass is Long Interspersed Nuclear Elements (LINEs), which also code for pol and gag ORFs, allowing for autonomous movement. However, LINEs do not encode an envelope protein and cannot move horizontally. Finally, the Short Interspersed Nuclear Elements (SINES) are non-autonomous elements that are dependent on LINE proteins to move [10].

Class II DNA transposons use a “cut and paste” transposition mechanism [9] (Figure 1b). DNA transposons encode a transposase, which recognizes the terminal inverted repeats of the transposon. An encoded transposase catalyzes cleavage and removal of the element from the original genomic location and insertion into a new genomic location [14]. This results in a double-stranded DNA break at the original insertion site but does not increase the copy number. One proposed method for DNA transposons to increase their copy number is by transposition from downstream of a replication fork to a position upstream of a DNA replication fork [4]. This class includes P-elements, which invaded wild *D. melanogaster* and are currently invading the sibling species *Drosophila simulans* [15,16]; the Ac/Ds elements in maize initially described by Dr. McClintock [1]; and dTc1 elements in *Caenorhabditis elegans* [17]. Helitrons are a second type of DNA element that moves via a circular single-stranded DNA intermediate [18]. This intermediate is proposed to invade the genome via a nick in the target DNA sequence followed by strand invasion. This mechanism creates one and a half copies of the original transposon, and replication or repair is proposed to resolve the missing half. A third type of DNA element is called Mavericks/Polinton*,* and the mechanism of movement is still under investigation [14,19,20]. Typically, Polintons are 15–25 kilobase long, which is much larger than other transposons, and they encode many proteins with homology to DNA polymerase and integrase [14,21]. In *Caenorhabditis briggsae*, Mavericks transposons have recently been implicated in horizontal gene transfer between different nematode species [22].

### 1.2. Transposons … Good or Bad?

Transposons were initially thought of as purely “selfish” elements, but it is now clear that the host can benefit from transposition-induced genetic variation and transposon genes can be directly co-opted or domesticated for host functions [3,23,24]. For example, transposable elements can carry regulatory sequences that are dispersed throughout the genome and can regulate gene networks. Different transposon families also have different insertion site preferences [25,26], which may allow the host to use co-opted elements to activate regulatory networks by separate mechanisms in distinct cell types [23,27]. Many species also contain genes that are derived from transposons. For example, telomerase is essential to chromosome maintenance and appears to have been derived from a retrotransposon reverse transcriptase [28]. Transposon-derived genes function in a variety of additional pathways, indicating that “domestication” has occurred multiple times throughout evolution [14]. Intriguingly, *Drosophila* lacks telomerase [29] and instead has co-opted the HeT-A, TART, and TAHRE retrotransposons, which cap the ends of chromosomes [29,30,31,32].

The examples above show how transposons have benefited the host, but uncontrolled transposon mobilization causes single- and double-stranded DNA breaks, producing genome instability [33]. Additionally, transposon insertions into essential coding regions or regulatory elements produce deleterious gene mutations [9,33]. Maintaining an intact genome is important for cell survival, but is especially critical for germ cells, which are dedicated to transmission of an intact genome to the subsequent generation. The small silencing piRNAs have a conserved role in suppressing transposons in germline cells [34] (reviewed in [35,36]). 

## 2. piRNA Pathway

### 2.1. Discovery of piRNAs and the piRNA Pathway

piRNAs were first discovered in studies of the *Suppressor of Stellate* (*Su[Ste])* locus on the *Drosophila* Y chromosome, which produced small RNAs that silenced the *Stellate* (*Ste*) gene repeats on the X chromosome [37]. Mutations in *Su[Ste]* are associated with male infertility and result in a loss of small RNAs targeting *Ste* repeats, the overexpression of Stellate protein, and the formation of Stellate crystals in the testes [38,39]. Subsequent cloning and sequencing of small RNAs from different tissues and stages of *Drosophila* development revealed small RNAs that mapped to additional repetitive regions, and most prominently, transposable elements [40]. This gave rise to the name “repeat-associated small interfering RNAs” (rasiRNAs) and suggested a role in silencing transposable elements (TE). 

At 23–26 nucleotides long, the rasiRNAs were known to be longer than the previously described 21-nucleotide-long micro RNAs (miRNAs) and small interfering RNAs (siRNAs) [40]. However, it was not initially clear if they were produced by a different biogenesis pathway, or if they were bound by the same family of proteins. miRNAs and siRNAs are produced by cleavage of double-stranded precursor RNAs by Dicer and are bound by AGO Argonaute proteins [41,42,43,44]; and in 2006, it was shown that rasiRNA biogenesis does not require Dicer and that is bound to PIWI clade Argonaute proteins [34,45]. In addition, a significant fraction of the small RNAs that co-precipitate with PIWI proteins in mammalian testes do not map to repeats [46,47,48,49,50], and the rasiRNAs were therefore renamed Piwi-interacting RNAs (piRNAs).

Piwi, the founding member of the PIWI clade Argonaute proteins, was discovered in a screen for genes required for germline stem division and maintenance [51,52]. Many other piRNA pathway genes, including *aub*, *vasa*, *cuff*, and *zuc*, were first identified in screens for mutations that disrupt *Drosophila* embryonic patterning and female fertility [53,54,55]. Significantly, mutations leading to genome instability in the female germline activate ATR/Checkpoint kinase 2 (Chk2) DNA damage signaling, which in turn disrupts embryonic patterning [56,57]. As a result of this discovery, previous screens for mutations that disrupt embryonic patterning and female fertility were used to rapidly identify numerous components of the piRNA pathway [53,54,58]. Later, screens using RNAi and directly assaying for transposon overexpression added to the growing list of piRNA pathway genes [59,60,61]. The combination of genetics, small-RNA deep sequencing, and subcellular localization studies in *Drosophila* led to rapid progress toward defining piRNA biogenesis and function [35,62,63].

### 2.2. Source of piRNAs

The most abundant piRNAs are derived from piRNA clusters, which are defined by the density of unique piRNA reads [64]. In *Drosophila*, these clusters are primarily found in pericentromeric and sub-telomeric heterochromatic regions on all four chromosomes and consist of nested transposon fragments [64,65]. These loci are thought to provide genetic memory of past transposon invaders [65] that produce small RNAs that repress surviving copies of transposon insertions [66]. However, it is now clear that isolated transposon insertions can also produce piRNAs [67,68,69]. The processing of transcripts from isolated transposons into small RNAs is thought to facilitate effective TE silencing [68]. However, piRNAs originating from the isolated TEs are much lower in abundance relative to piRNA clusters, and most isolated TE insertions do not make piRNAs [69]. What designates some insertions as piRNA-producing and their exact role in TE silencing are still unclear. 

As noted above, *Drosophila* telomeres are comprised of HeT-A, TART, and TAHRE retrotransposon repeats [29,70]. Transcription of these arrays is bidirectional and initiated from transposon promoters [70]. There is evidence that piRNAs produced from these transcripts directly regulate the activity of HeT-A, TART, and TAHRE and contribute to telomere maintenance [70,71]. This suggests that piRNAs regulate “domesticated” TEs in addition to “selfish” ones.

*D. melanogaster* clusters are classified as “uni-strand” or “dual-strand”, based on the strand biases of the encoded piRNAs [66]. Uni-strand clusters are predominantly active in the somatic follicle cells that surround the germline in the ovaries and produce piRNAs from one genomic strand [66]. Transcription of uni-strand clusters produces capped, spliced, and polyadenylated precursors that are structurally similar to canonical protein-coding gene transcripts [67,72,73]. The best studied uni-strand cluster is the *flamenco (flam)* locus [73]. Transposon fragments in this cluster are strongly anti-sense-biased relative to the direction of transcription. Thus, processing *flam* precursors produces piRNAs that are anti-sense to the embedded transposon sequences [72,73]. The *flam* locus is enriched for fragments of the *gypsy* retrotransposon, and promoter mutations that block transcription and piRNA production lead to *gyspy* overexpression and sterility [64,74]. Transposon insertions into *flam* also appear to be relatively recent and potentially acquired through horizontal transfer [73], indicating that *flam* is actively engaged in the host response to the transposon invasion of the somatic follicle cells.

Dual-stranded clusters, on the other hand, are expressed in germline nurse cells, produce precursors, and piRNAs from both genomic strands, and carry randomly oriented transposon fragments [64]. The dominant dual-strand cluster in *Drosophila* is cytologically located at 42AB, near the centromere on Chromosome 2R. The 42AB cluster is over 250 kilobases long, consists primarily of nested transposon fragments, and produces approximately 30 percent of the piRNAs in the female germline [64]. Surprisingly, the deletion of this cluster does not compromise germline survival, transposon silencing, or fertility [75], suggesting that piRNAs derived from other smaller clusters or isolated insertions compensate for the deletion. However, this cluster also appears to target older elements that have largely degenerated and are no longer active. The cluster may therefore be dispensable under laboratory conditions but provide a genetic memory of past genome invaders and thus prevents re-invasion by related elements. While the major dual-strand cluster is dispensable for fertility, mutations that significantly reduce germline piRNA production lead to infertility and germline genome instability. The remainder of this review will focus on the germline piRNA pathway and the potential regulation of this pathway in response to stress.

### 2.3. Germline piRNA Precursor Expression and Nuclear Export

**Figure 2:**
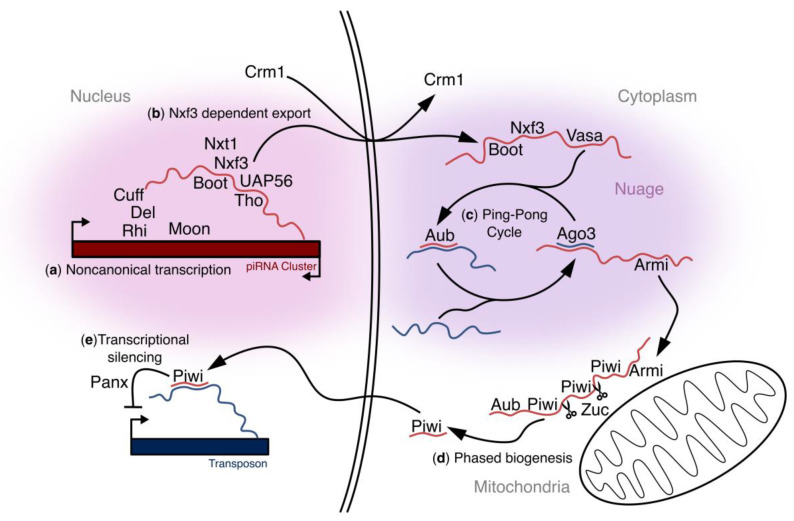
*D. melanogaster* germline piRNA biogenesis pathway: (**a**) Non-canonical transcription: piRNA clusters are bound by Rhino (Rhi). Rhino interacts with Deadlock (Del), and this helps recruit Moonshiner (Moon) to piRNA clusters. Moon recruits transcriptional machinery to promote non-canonical transcription. Del interacts with Cutoff (Cuff) and Cuff is thought to protect the 5ʹ end of cluster transcripts. (**b**) Nxf3-dependent export: Bootlegger (Boot) and the Tho complex (Tho) bind piRNA precursors and recruit UAP56 binding. Boot also helps recruit the Nxf3-Nxf1 hetero-dimer, which promotes the export of the piRNA-cluster transcripts in a Crm1-dependent manner. All the components involved in non-canonical transcription and Nxf3-dependent export colocalize at distinct foci within the nucleus. (**c**) Ping-Pong cycle: Transcripts exported into the cytoplasm are bound by Boot, Nxf3, and Vasa. These transcripts are targeted for cleavage by Ago3. Cleavage products are then loaded into Aub, which can target transposon transcripts for cleavage. These cleavage products are subsequently loaded into Ago3, which can target cluster transcripts to initiate the Ping-Pong cycle once again. Ago3 cleavage products are also bound by Armitage (Armi) and brought to the outer mitochondrial membrane for phased piRNA biogenesis. Boot, Nxf3, Vasa, Aub, Ago3, and Armi all colocalized to perinuclear nuage granules. (**d**) Phased biogenesis: Aub bound to Ago3 cleavage products are shuttled to the outer mitochondrial membrane with the help of Armi. Zucchini (Zuc) cleaves the transcripts to release Aub. Subsequent Piwi loading followed by Zuc cleaving events occur down the length of the transcript, producing “phased” piRNAs, and Armi is proposed to facilitate this process. Zuc and Armi are localized to the mitochondria. (**e**) Transcriptional silencing: Piwi localizes to the nucleus and binds to transposon transcripts co-transcriptionally. Piwi recruits proteins involved in laying down H3K9me3 marks to help silence transposons. This process requires Panoramix (Panx).

Dual-stranded piRNA clusters are generally found at the boundary between heterochromatic and euchromatic regions of the genome and are marked with H3K9me3, which is associated with transcriptional silencing [64]. piRNA-cluster transcription relies on a non-canonical process initiated by the binding of Rhino, an HP1 homolog [67] (Figure 2a). Like other HP1 family proteins, Rhino contains a chromodomain that binds specifically to H3K9me3, a shadow domain that is required for protein-protein interactions, and a hinge domain that acts as a linker [67,76,77,78,79]. Rhino binds to H3K9me3 on dual-strand piRNA clusters and recruits Deadlock (Del), a rapidly evolving protein that contains no known domains, and Cutoff (Cuff), a Rail1-like protein that is proposed to bind to the cap on piRNA-cluster transcripts [58,67]. These three proteins form the Rhino–Deadlock–Cutoff complex (RDC), which marks dual-stranded clusters [58,67,80,81] and is rapidly evolving [76,82].

Rhino, bound to its partner Deadlock, recruits a non-canonical transcription initiation complex consisting of Moonshiner (Moon), Transcription initiation factor IIA subunit 2 (TFIIA-S), and TATA box-binding protein-related factor 2 (TRF2). This complex promotes transcription initiation from both strands throughout the cluster, independent of defined promoter elements [83]. Additionally, the deletion of canonical gene promoters and promoter-like regions flanking the 42AB cluster does not block cluster transcription [83]. Chang, Mattei [84] showed that canonical transposon promoters within dual-stranded clusters are actively repressed by the protein Maelstrom (Mael), which is proposed to facilitate Rhino-dependent non-canonical transcription. Dual-strand piRNA clusters thus rely on non-canonical transcription to produce piRNA precursors from both genomic strands.

The RDC complex has also been implicated in the suppression of splicing, producing long un-spliced precursor transcripts [81]. Tethering a LacI:Rhino fusion protein to *LacO:GFP* reporter transgene, which normally produces spliced transcripts, resulted in the suppression of splicing. The mechanism by which RDC suppresses splicing is unknown. However, Cutoff is a homolog of the de-capping enzyme Rai1 but lacks key catalytic residues [81,85,86]. Cutoff binding to capped cluster transcripts could therefore block binding by the Cap Binding complex, which promotes splicing.

The export of un-spliced cluster transcripts relies on a non-canonical export pathway (Figure 2b). The RDC recruits Bootlegger (Boot) and the THO complex to cluster transcripts [87,88,89,90], and Bootlegger and the THO complex promote binding of the DEAD box RNA-binding protein Uap56 [88,90]. Uap56, together with the THO complex, forms the transcription and export complex (TREX), which functions with Bootlegger to hand off precursors to nuclear export factor 3 (Nxf3) and Nxt1. Nxf3 contains nuclear export signals recognized by Chromosomal Maintenance 1 (Crm1) or Exportin 1, a protein involved in the nuclear export of large molecules including RNA and proteins, and Crm1 is found in yeast and humans [91,92,93]. The Nxf3–Nxt1 complex thus mediates the Crm1-dependent nuclear export of piRNA precursors [89,90,94].

Once exported into the cytoplasm, piRNA precursors are processed into small silencing RNAs, while mRNAs are loaded on ribosomes for translation into proteins. Maintaining the fidelity of transcript sorting is critical, and Uap56 and Nxf3 appear to have key functions in this process. Un-spliced cluster transcripts preferentially co-precipitate with Uap56 and the THO complex [88]. The THO complex is a heteropentomer composed of THO2, HRP1, THOC5, THOC6, and THOC7. HPR1 and THOC2 are conserved from yeast to humans and form a core complex, and mutations in the cognate genes are lethal. Homozygous null alleles of *uap56* are also lethal, but a null allele combined with a point mutation in a conserved surface residue required for wild-type binding to THO is viable but sterile and defective in transposon silencing and piRNA biogenesis [88,95]. Similarly, mutations in *Drosophila thoc5* and *thoc7* are viable but sterile and disrupt piRNA biogenesis [87,88]. The core THO complex and UAP56 are therefore essential, while the intact TREX appears to be particularly critical to piRNA-cluster transcript nuclear export [88]. Nxt1 can form a hetero-dimeric complex with Nxf3 or Nxf1 and the Nxf1-Nxt1 is required for mRNA export [90,96]. By contrast, TREX functions upstream of Nxf3-Nxt1, which is required for piRNA precursor export [89,90]. The TREX and Nxf3-Nxt1 thus function in a pathway that distinguishes piRNA precursors from mRNAs. 

### 2.4. Ping-Pong Biogenesis

**Figure 3:**
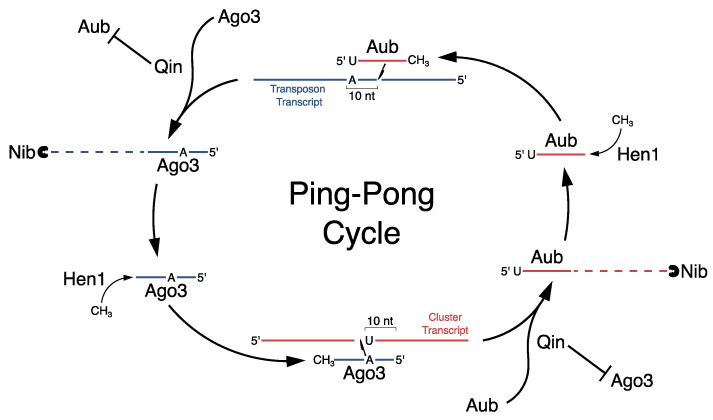
Steps of the Ping-Pong cycle: Mature piRNAs loaded into Aub have a 5′ uridine bias and can target transposon transcripts for cleavage between nucleotides complementary to the piRNA at positions 10 and 11. Qin is proposed to maintain the fidelity of Ago3 binding to the transposon cleavage product. Subsequent trimming and methylation by Nibbler (Nib) and Hen1 respectively produced a mature Ago3-loaded piRNA complex. This complex can then target cluster transcripts for cleavage. The fidelity of Aub loading onto cleaved cluster transcripts is also proposed to require Qin. Nib and Hen1 then facilitate the maturation of Aub-loaded piRNAs to complete the cycle.

After cluster transcripts are exported from the nucleus, two pathways drive piRNA biogenesis. The Ping-Pong cycle amplifies the piRNA pool (Figure 2c), and phased biogenesis increases piRNA sequence diversity (Figure 2d). Most of the proteins required for Ping-Pong biogenesis localize to perinuclear granules known as nuage, including the DEAD box RNA-binding protein Vasa; the PIWI clade Argonautes Aub and Ago3; and Tudor domain-containing proteins like Krimper, Kumo, and Qin [64,97,98,99]. In the Ping-Pong cycle, Aub, loaded with initiator piRNAs derived from cluster transcripts, cleaves complementary sense-strand transposon transcripts, leading to post-transcriptional silencing (Figure 3). This also generates a 5′ cleavage product that is bound by Ago3, forming the precursor of the responder piRNA. Subsequent trimming of the 3ʹ end by the exonuclease Nibbler and methylation by Hen1 creates a mature “sense” piRNA [100,101]. The loaded Ago3 can then become the new initiator piRNA and target cluster transcripts for cleavage and subsequent loading into Aub. Again, trimming and methylation by Nibbler and Hen1, respectively, drive the maturation of the responder piRNA. Aub, loaded with anti-sense piRNAs, continues the cycle [64,102]. 

The Ping-Pong model is supported by the mechanism of piRNA-guided cleavage and the relationship between piRNAs from opposite strands [64,102]. PIWI proteins catalyze target cleavage between nucleotides 10 and 11 of the piRNA guide. The cleavage product carrying a free 5′ end thus overlaps with the guide by 10 nucleotides (nt), and piRNAs from opposite strands or initiator and responder piRNAs show a strong bias toward a 10nt overlap. piRNAs loaded into Aub are anti-sense-biased relative to transposons, while piRNAs bound by Ago3 show a strong sense-strand bias, and together, piRNAs bound by these proteins show a particularly strong 10nt overlap. In addition, *ago3* mutants lead to a collapse in this strand bias [103], strongly supporting piRNA production through reciprocal cleavage events [64,102]. The statistical significance of this 10nt overlap bias, expressed as a z-score, is a common measure of the efficiency of the Ping-Pong cycle. 

piRNAs that are anti-sense to transposable elements are predominantly loaded into Aub, but the molecular mechanism that generates this bias is not fully understood. Maternally deposited Aub loaded with anti-sense piRNAs could drive sense piRNAs into Ago3 via the Ping-Pong cycle, perpetuating the bias [60,66]; however, this does not explain how the bias was initiated. Mutations in the genes encoding the Tudor-domain proteins Qin and Krimper, which localized to the nuage, cause a loss of strand bias in Aub and Ago3, suggesting that strand bias is actively enforced by these proteins [98,104,105,106]. This loss of bias is observed in adult females, and these females developed from embryos that inherited maternal Aub carrying anti-sense-biased piRNAs. Qin and Krimper are therefore required to maintain the inherited strand bias, but it is unclear if they are also required to establish the bias. 

In addition to the proteins mentioned previously, nuage contains multiple other proteins involved in piRNA processing and the Ping-Pong cycle. Vasa is a deeply conserved piRNA biogenesis protein and nuage component [66,107,108]. This DEAD box protein is required for Ping-Pong amplification, binds piRNA-cluster transcripts [95], and appears to facilitate the removal of cleaved substrates from Piwi–piRNA complexes, allowing for multiple turnovers [109]. A number of Tudor domain-containing proteins are also required for piRNA biogenesis and bind to demethylated arginines on Aub and Ago3, consistent with a proposed scaffolding function that may help organize the Ping-Pong machinery [98,99,109,110]. 

### 2.5. Phased piRNA Biogenesis

**Figure 4:**
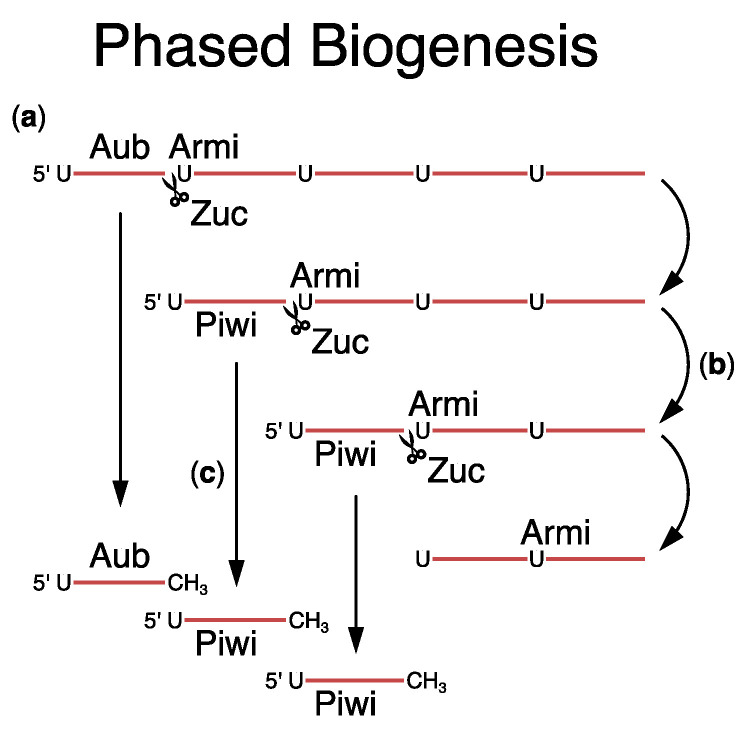
Steps of phased piRNA biogenesis: (**a**) Aub bound to a long piRNA-cluster transcript is the precursor for phased biogenesis. Armi is proposed to shuttle this Aub-bound cluster transcript from the nuage to the mitochondria, where Zuc then releases Aub by cleavage at the downstream uridine. This creates the 5′ end of the next piRNA. (**b**) Piwi is loaded onto the new 5′ end of the cluster transcripts. Zuc cleavage at the downstream uridine releases Piwi and creates the 5′ end of the subsequent piRNA. This process can continue down the length of the cluster transcript. (**c**) Further trimming and methylation produce mature Piwi-bound piRNAs that spend the length of the piRNA-cluster transcripts.

Piwi is the founding member of the PIWI clade Argonaute proteins and is the third and final member found in *D. melanogaster* [51]. Piwi is loaded with trail piRNAs through the phased biogenesis pathway, which is initiated when an Ago3-cleaved cluster transcript is loaded onto Aub, producing a long precursor bound to Aub [106,111] (Figure 4a). Armi, an RNA binding ATPase, localizes at nuage and mitochondria and is proposed to shuttle Aub-bound precursors from the nuage to the mitochondria [111]. The mitochondrial endonuclease Zucchini cleaves the Aub complex from the precursor. Trimming then generates a mature Aub–piRNA complex. Zucchini cleavage generates fragments with a 5′ uridine, which is bound by Piwi to form the precursor for the next trail piRNA [106,111,112,113] (Figure 4b). Subsequent cycles of Zucchini cleavage, 3′ trimming, and methylation produce head-to-tail arrays of mature piRNAs bound to Piwi (Figure 4c). Processive processing through the phased biogenesis pathway is proposed to diversify the piRNA pool, while the Ping-Pong cycle drives piRNA amplification.

### 2.6. piRNA Mediated Transposon Silencing

piRNA-mediated transposon silencing occurs in both the cytoplasm and the nucleus. Post-transcriptional transposon silencing occurs through the Ping-Pong cycle. Aub and Ago3 are cytoplasmic and drive Ping-Pong amplification, and their slicer activity is required for transposon silencing [64,102]. piRNAs loaded into Aub are complementary to transposon transcripts and guide post-transcriptional silencing through target cleavage. In contrast, Piwi is nuclear and does not require catalytic activity to direct silencing [114,115,116] (Figure 2e). The nuclear localization of Piwi requires piRNA loading, and mutants in Piwi that remove the nuclear localization signal (NLS) fail to silence transposons [116].

The piRNAs that are loaded into Piwi are anti-sense-biased relative to transposon transcripts and are thought to guide association with nascent transcripts. Piwi then initiates silencing by recruiting factors that drive transcriptional silencing, including Asterix/Gtsf1 (Arx), the PANDAS/SFiNX/PICTS complex, and Maelstrom [60,117,118,119]. Gtsf1/Asterix (Arx) is thought to trigger a Piwi conformation that is permissive for binding the downstream silencing machinery [60,118,120,121]. By contrast, other orthologues of Gtsf1 enhance the target cleavage activity of catalytically active PIWI Argonaute proteins [121]. 

The PANDAS/SFiNX/PICTS complex is composed of Panoramix/Silencio (PanX) Nxf2, Nxt1, and LC8/Cut-up [119,122,123,124,125]. Nxf2 is a paralog of the RNA export factor Nxf1 but promotes transcriptional silencing [123,125,126,127]. Tethering Panoramix to RNA is sufficient to induce transcriptional silencing and is thought to recruit multiple downstream chromatin modifiers to drive heterochromatin formation [119,122]. These recruited factors include LSD1, which removes the active mark H3K4me2/3 [128], and Setdb1/Eggless, which deposits the silencing mark H3k9me3 [129]. HP1a binds to H3k9me3 and recruits SetDb1, which modifies neighboring nucleosomes, spreading the silencing mark to flanking regions [119]. By directing the PANDAS/SFiNX/PICTS complex to transposons, Piwi is therefore proposed to promote repressive chromatin modifications that transcriptionally silence targets. Finally, Maelstrom represses canonical transcription at transposon insertions inside and outside clusters [84], and Not1-CcR4 interactions with Piwi have been implicated in the regulation of telomeric transposons [130]. However, the mechanisms underlying these functions remain to be determined.

### 2.7. Organization of the Pathway

Rhino localizes to distinct puncta throughout germline nurse-cell nuclei [58], and chromatin immunoprecipitation-sequencing (ChIP-seq) shows that Rhino binds specifically to dual-strand piRNA clusters, which are marked by H3K9me3 [67,81]. However, Rhino does not bind to many other chromosomal regions marked by H3K9me3, and how the RDC is restricted to clusters is not well understood. Maternally deposited piRNAs bound by Piwi have been proposed to guide initial Rhino localization, with the maintenance of localization through a piRNA-independent mechanism [67,131,132]. However, the depletion of Piwi early in embryo development results in decreased Rhino localization to clusters, but significant binding is retained [131]. Additionally, genetically removing the maternally deposited piRNAs that uniquely map to the 42AB cluster does not block Rhino binding or piRNA production from the paternally inherited 42AB cluster [133]. Kipferl, a zinc finger protein, and the THO complex also appear to restrict Rhino to the piRNA cluster but are not essential to Rhino localization [88,134]. Recently, siRNAs from repeated sequences have been implicated in the de novo assembly of piRNA clusters, which are established over multiple generations [135]. The siRNA and piRNA systems may therefore cooperate to define piRNA clusters, but this remains to be rigorously tested.

Deadlock, Cuff, and the TREX complex colocalize with Rhino at nuclear foci [67,87,95], suggesting that the production of piRNA precursors occurs in distinct nuclear compartments, which contain the precursor transcription and export machinery. Similarly, the Ping-Pong machinery localizes to perinuclear nuage granules [97,110]. Nuage is French for “cloud” and is classically defined as membrane-less, electron-dense, perinuclear structures [136,137]. In organisms ranging from sea urchins to humans, nuage is specifically found in the germ cells and is usually perinuclear or associated with the mitochondria [136]. While nuage composition and function were initially mysterious, it is now clear that key piRNA pathway components, including Vasa homologs and several Piwi proteins, localize to nuage in phylogenetically diverse systems [97,108,138,139,140]. However, fluorescence recovery after photobleaching (FRAP) experiments indicates that nuage-associated piRNA proteins are in rapid equilibrium with cytoplasmic pools [141]. It is therefore unclear if piRNA production and target silencing take place in nuage, the cytoplasm, or both compartments.

The nuclear and nuage piRNA biogenesis compartments also appear to be connected, as the location of perinuclear granules labeled with Vasa is highly correlated with Rhino foci at the nuclear periphery [95]. Additionally, mutations in *uap56* disrupt Vasa localization to nuage, and both Uap56 and Vasa bind to piRNA-cluster transcripts. These observations also imply that Uap56 is required for nuage formation and the spatial linkage between nuclear and cytoplasmic piRNA biogenesis compartments. Our recent studies confirm that Uap56 is required for the spatial linkage of clusters and nuage but also indicate that the loss of Vasa from nuage is secondary to the activation of Chk2 DNA damage signaling [142]. We speculate that the juxtaposition of nuclear and cytoplasmic compartments is the result of a handoff of piRNA-cluster transcripts from Uap56 to proteins in nuage; however, this model remains to be tested.

## 3. The piRNA Response to Novel Transposons

piRNAs silence established endogenous transposons but also silence recent transposon invaders. Hybrid dysgenesis is a sterility phenotype induced when males carrying a novel transposable element invader are crossed to naïve females [143,144]. This results in transposon activation in the offspring and germline DNA damage [143,145]. The two classic examples are I–R dysgenesis and P–M dysgenesis, which result from the introduction of the I-element retrotransposon and the P-element DNA transposon, respectively [16,145]. 

### P-Element Invasion and piRNA Adaptation

The P-elements swept through the wild *D. melanogaster* in the 1900s, after early lab strains were isolated [144]. Older lab strains are therefore “naïve” to the P-element transposon. P–M hybrid dysgenesis occurs when naïve lab-strain females are crossed to wild males containing the P-element. This leads to P-element activation and decreased fertility or sterility in the F1 generation. The reciprocal cross, where the P-element is introduced through wild females, produces fertile F1 progeny. The directionality of hybrid dysgenesis suggests that wild females deposit a “factor” that helps maintain fertility, and Brennecke, Malone [132] showed that this factor is piRNAs mapping to the P-element.

P–M dysgenesis results in F1 adult progeny that are initially sterile, but Khurana, Wang [146] found that F1 adult females are initially sterile, but gradually regain fertility as they age. The initial sterility correlated with activation of P-elements, overexpression of some endogenous transposons, and disruption of piRNA pathway organization. Recovery of fertility, by contrast, correlates with restoration of the piRNA pathway organization, production of P-element mapping piRNAs, and novel insertion of the P-element into a piRNA cluster. It was proposed that the insertion of this transposon into a cluster allows for heritable adaptation. However, the F1 progeny inherit the P-element insertions that are present in wild flies, which silence this element. The onset of silencing could be due to activation of piRNA biogenesis from one or more of the inherited insertions. Supporting this model, isolated transposable element insertions can produce piRNAs [67], suggesting that transposon insertion into a cluster may not be essential to the piRNA response.

The fertility defects observed with P–M dysgenesis are sensitive to temperature, as dysgenic flies raised at 18 °C are fertile, while dysgenic flies raised at 25 °C are sterile [144,147,148]. Consistent with this observation, Moon, Cassani [148] found that dysgenic flies raised at 25 °C accumulated higher levels of P-element insertions than flies raised at 18 °C. Intriguingly, the recovery of fertility in dysgenic females requires Checkpoint kinase 2 (Chk2), a signaling protein required to propagate the DNA damage response [148,149]. Moon, Cassani [148] proposed that P-element mobilization and the resulting DNA damage activates signaling through Chk2, which induces developmental arrest that provides the host time to repair the damage and initiate piRNA production against the invading P-element. This coordinated response is proposed to allow for adaptation to newly invading transposons [148].

## 4. The piRNA Pathway Is Modulated by the External Environment and Stress

Through studies of hybrid dysgenesis, it has become clear the piRNA pathway is not static. When challenged with a novel P-element, the piRNA pathway is able to respond and adapt through the production of P-element mapping piRNAs [146]. It is becoming evident that temperature and DNA damage signaling can also modulate the piRNA pathway.

### 4.1. Intersection between DNA Damage Signaling and the piRNA Pathway

The ATM-Chk2 kinase system coordinates cellular response to DNA damage, leading to developmental arrest, the initiation of DNA repair, or the activation of p53-dependent apoptosis [149,150,151]. Essentially all piRNA pathway mutations activate transposons, leading to genome instability and germline DNA damage. However, many *Drosophila* piRNA mutations also disrupt asymmetric localization of key morphogenetic RNAs during oogenesis, and this process defines the anterior–posterior and dorsal–ventral axes of the embryo [54]. The implication is that piRNAs control transposons and genes in the axis-specification pathway. However, the axis-specification defects associated with *Drosophila* piRNA mutations were suppressed by a null mutation in *mnk*, which encodes the *Drosophila* Chk2 homolog [57,85,152,153]. During *Drosophila* oogenesis, therefore, piRNAs appear to be dedicated to transposon silencing, and the axis-specification defects are secondary to DNA damage signaling. By contrast, the recovery of piRNA-guided P-element silencing during hybrid dysgenesis is blocked by *mnk* mutations [148]. As noted above, this could reflect a requirement for Chk2-mediated developmental cell cycle arrest, allowing time for recovery, but Chk2 could have a more direct role in regulating piRNA biogenesis.

Intriguingly, the analysis of an unusual allele of *rpp30*, which encodes a subunit of the tRNA processing enzyme RNase P, suggests that Chk2 activation can inhibit the piRNA pathway, enhancing genome instability. RNase P is required for tRNA maturation, and null alleles are lethal, but Molla-Herman, Valles [154] identified a hypomorphic *rpp30* allele that leads to female sterility, the collapse of piRNA production, and transposon upregulation. Remarkably, all these defects, including sterility, are suppressed in double mutants with *mnk* [154]. In addition, systematic comparisons of single piRNA mutants with corresponding *mnk* double mutants indicate that Chk2 activation displaces Vasa and Aub from nuage granules [142]. How Chk2 regulates nuage composition is still unclear, but piRNA mutants trigger the Chk2-dependent phosphorylation of Vasa [57]. However, the function of Vasa phosphorylation has not been established. 

Data on the *rpp30* mutants indicate that Chk2 activation can have a profound impact on oogenesis, but piRNA mutants activate Chk2 and generally do no block oogenesis. In addition, Vasa and Aub localization to nuage is restored when *mnk* is combined with several different piRNA mutants, but this is associated with only subtle changes in piRNA expression and transposon silencing [142]. This apparent discrepancy likely reflects when *rpp30* and piRNA mutants impact oogenesis. Female mutants for *rpp30* have rudimentary ovaries and do not produce eggs, indicating that the gene is required at an early step in oogenesis [154]. piRNA mutations, by contrast, do not block oogenesis, and mutant females produce embryos that fail to hatch, indicating a function in later stages of oocyte development. These findings imply that *rpp30* mutations activate Chk2 DNA damage signaling early in oogenesis, blocking development, while piRNA mutations lead to genome instability much later, when Chk2 may fine-tune the piRNA pathway function. The delayed impact of piRNA mutations on oogenesis is likely due to the maternal deposition of piRNAs and PIWI proteins, which may support oogenesis through larval and pupal stages.

These findings clearly demonstrate the impact of damage signaling on the biological phenotypes of piRNA pathway mutations and indicate that mutations that disrupt functions outside this pathway that lead to genome instability can produce secondary defects in piRNA biogenesis. It is therefore critical to consider the potential impact of DNA damage signaling when interpreting the phenotypes associated with mutations leading to genome instability. 

### 4.2. A DNA Damage Amplification Circuit?

Mutations in the *Drosophila* piRNA pathway mobilize transposons, leading to DNA damage and Chk2 damage signaling. Chk2 activation then appears to further compromise the piRNA pathway, enhancing transposon mobilization. This feed-forward amplification of genome instability would appear to be highly detrimental, particularly in the germline. However, a DNA damage amplification loop could help ensure that only intact genomes are passed to the next generation by triggering a suicide response that eliminates oocytes carrying compromised genetic material. Alternatively, McClintock proposed that genomic stress activates transposons in order to generate potentially beneficial mutations that may increase offspring survival [155]. The relatively modest impact of Chk2 activation on the piRNA pathway in adult *Drosophila* could allow levels of transposition that would not block oogenesis and could support the generation of potentially beneficial mutations. 

### 4.3. Temperature and Heat Stress Modulates the piRNA Pathway

There is growing evidence that the piRNA pathway is sensitive to temperature. *D. melanogaster* raised at 29 °C show increased Ping-Pong processing, and presumably transposon silencing, relative to flies raised at 18 °C [156]. How elevated temperatures increase Ping-Pong efficiency is unclear, but the authors suggest that higher temperatures may relax the RNA structure to make targets more accessible to piRNAs. Additionally, piRNAs are produced from a cluster of P-element-derived transgenes in flies raised at 29 °C, while this cluster is quiescent in flies raised at 25 °C [157]. It is unclear how temperature activates piRNA production, or how the piRNA pathway senses temperature.

The response of protein-coding genes to heat stress is well characterized. Upon heat shock, transcription is globally repressed, but the expression of a subset of heat shock proteins (hsp) is rapidly induced [158]. Hsps are conserved chaperone proteins that prevent protein aggregation and promote protein folding and stability. Hsp70 and Hsp90 are needed to load duplexes into Argonaute proteins for miRNA and siRNA biogenesis [159,160], and Hsp83 (Hsp90) has been implicated in piRNA loading into PIWI clade Argonautes [161,162]. Heat shock has also been reported to downregulate some piRNA clusters, with a slight decrease in piRNA abundance [163], and to induce Ago3 degradation [164]. Heavy heat stress thus appears to compromise piRNA biogenesis, potentially activating transposons. 

We have recently analyzed the impact of heat stress on the subcellular organization and function of the piRNA pathway. With a short, acute heat shock, Rhino is displaced from clusters, but localization recovers after flies are returned to control temperatures [133]. The loss of Rhino localization correlates with decreased cluster transcript expression, consistent with Rhino’s role in initiating non-canonical transcription [83]. In addition, heat shock disrupts Vasa and Aub localization to the perinuclear nuage [133]. piRNA pathway organization is therefore sensitive to heat stress, which may modulate downstream transposon control in laboratory settings. Flies in the wild encounter environmental temperature changes/extremes, and it is possible that these environmental changes impact piRNA biogenesis or functions in transposon suppression. 

### 4.4. The Intersection between Stress, Transposons, and piRNAs

In *D. melanogaster*, various types of stress activate subsets of transposon families, but no one type of stress activates the majority of transposon families (reviewed in [165]). For example, ionizing radiation upregulates 10% of transposon families in whole male flies, and the number of transposon families upregulated correlates with the doses of radiation [166]. By contrast, in adult females, Cisplatin treatment, which also causes DNA damage, increases the expression of only four transposon families [167]. Milyaeva, Kukushkina [168] also reported that oxidative stress increases the expression of some LTR transposons and some piRNA clusters. Transposon silencing and the piRNA response to stress thus appear to vary with the type of stress and tissue. Whether other environmental factors affect the piRNA pathway remains mostly unexplored, but we speculate that the piRNA response to other stress may also be unique. The identification of these responses would also provide multiple ways to manipulate the piRNA pathway and potentially several tools for the investigation of the pathway itself. Determining the mechanisms leading to variability in transposon and piRNA response is key to understanding the impact of environmental changes on genome organization and function.

## 5. Concluding Remarks

Studies on *Drosophila* have provided remarkable insights into piRNA biogenesis and piRNA-mediated transposon silencing. An increasing body of work suggests that the piRNA pathway is “tuned” to environmental signals. McClintock [155] proposed that stress-induced transposon activation provides beneficial genetic diversity, driving adaptive evolution. The link between the germline transposon-silencing system and stress may provide a molecular foundation for this response.
